# Marine natural products: potential agents for depression treatment

**DOI:** 10.3389/abp.2024.12569

**Published:** 2024-04-30

**Authors:** Xunqiang Wang, Cece Yang, Xing Zhang, Caiping Ye, Wenping Liu, Chengmin Wang

**Affiliations:** Department of Psychiatry, Shenzhen Longgang Center for Chronic Disease Control, Shenzhen, China

**Keywords:** depression, marine natural products, neurotransmitter systems, synaptic plasticity, anti-inflammatory

## Abstract

Depression is a common psychiatric disorder. Due to the disadvantages of current clinical drugs, including poor efficacy and unnecessary side effects, research has shifted to novel natural products with minimal or no adverse effects as therapeutic alternatives. The ocean is a vast ecological home, with a wide variety of organisms that can produce a large number of natural products with unique structures, some of which have neuroprotective effects and are a valuable source for the development of new drugs for depression. In this review, we analyzed preclinical and clinical studies of natural products derived from marine organisms with antidepressant potential, including the effects on the pathophysiology of depression, and the underlying mechanisms of these effects. It is expected to provide a reference for the development of new antidepressant drugs.

## Introduction

As one of the most common mental illnesses, there are currently at least 50 million patients with depression in China (Huang et al., 2019). The incidence of depression is increasing year by year, and it is expected to become one of the major causes of disease burden in China by 2030 (Huang et al., 2019). Depression imposes a heavy burden on individuals, families and society due to its high incidence, high disability rate and high suicide rate (Cipriani et al., 2018). Selective serotonin reuptake inhibitors (SSRIs), norepinephrine reuptake inhibitors (NRIs), and dopamine reuptake inhibitors (DRIs) are the main first-line antidepressants used in clinical practice today (Harmer et al., 2017; Moragrega and Ríos, 2021). These medications either work on the neurotransmitter systems of serotonin (5-HT), Norepinephrine (NE), and dopamine (DA) or they suppress the action of the enzyme monoamine oxidase (MAO) to produce antidepressant effects (Harmer et al., 2017). However, even after adequate and sufficient antidepressant treatment, about one-third of patients still do not have a significant therapeutic effect. Patients often experience side effects such as gastrointestinal discomfort and loss of libido, in addition to poor treatment compliance. Therefore, it is of great significance to find more effective and safer antidepressant compounds from a wide range of natural products, and it can also provide new ideas for the development of antidepressant products.

The living environment of marine organisms is complex, resulting in a large number of marine natural products (MNPs). Studies have shown that MNPs have significant pharmacological activities, and the toxicity and side effects are significantly lower than those of synthetic compounds (Corona, 2018). These marine-derived active substances have played an important role in the prevention and treatment of many diseases, and their pharmacodynamic mechanisms are constantly being elucidated. In recent years, with the relentless exploration of researchers, the bioactive substances derived from MNPs have increased. The metabolites isolated from marine organisms have diverse chemical structures, including polyketides, terpenoids, alkaloids, macrolides, cyclic peptides, quinones, polyethers, sterols, polysaccharides, unsaturated fatty acids, and a wide range of pharmacological activities, including antibacterial, antiparasitic, enzyme inhibitor, antioxidant, cytotoxic activity, etc. The majority of marine drug research and development is focused on anti-tumor, anti-cardiovascular disease, and antibacterial agents (Russo et al., 2015; Lima and Medeiros, 2022). Many marine compounds have received clinical approval for use, including the analgesic ziconotide and the anti-cancer drug cytarabine (Jimenez et al., 2020; Yang et al., 2021). MNPs and compounds generated from MNPs are becoming more and more valuable due to their biological activities. Numerous studies have recently revealed that MNPs have antidepressant properties and may slow the course of depression (Subermaniam et al., 2021a). MNPs may be a useful resource for the development of brand-new antidepressant alternatives. This article examines preclinical and clinical studies on the antidepressant effects of MNPs and research on the neuroscience of depression.

## Subsections relevant to the subject

### Pathogenesis of depression

**FIGURE 1:**
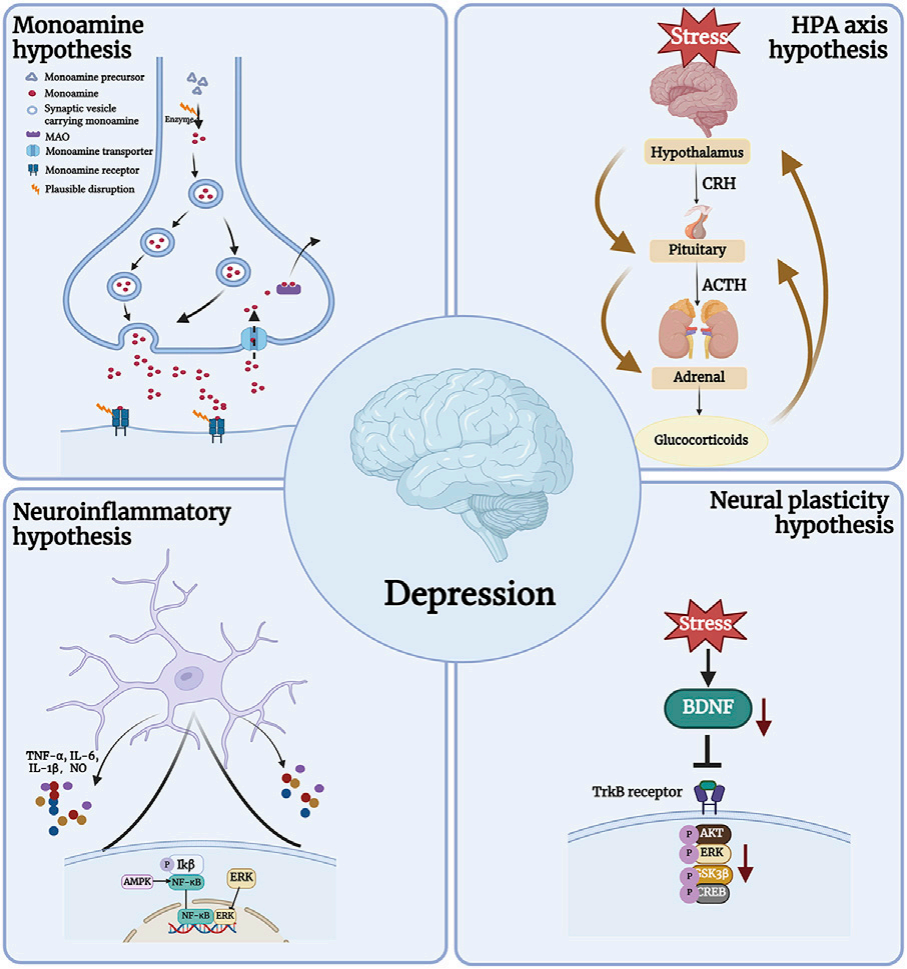
The pathogenesis of depression.

The pathogenesis of depression is complex and the biological mechanism is not fully understood (Jesulola et al., 2018). Currently, the widely accepted pathogenic hypotheses include the Monoamine hypothesis, the Neural plasticity hypothesis, the Neuroinflammatory hypothesis, and the Hypothalamic-pituitary-adrenal (HPA) axis (Figure 1) (Subermaniam et al., 2021a; Borbély et al., 2022). It should be mentioned that depression has a complex etiology and may be brought on by a confluence of various pathogenic variables. The search for drugs with multiple targets is thus a crucial area of research for the development of antidepressants.

### Monoamine hypothesis

One of the primary causes of depression has been determined to be decreased levels and functional deficiencies of 5-HT, DA, and NE, which are typically present in the brains of depressed patients (Matraszek-Gawron et al., 2019; Wang et al., 2022). The monoamine hypothesis is strongly supported by the fact that 5-HT or NERI can alleviate depression (Locher et al., 2017; Rana et al., 2021). By improving neurotransmission in the central nervous system and increasing the amount of related monoamine neurotransmitters in the synaptic cleft, this class of medications reduces the symptoms of depression (Locher et al., 2017; Yohn et al., 2017).

### Neural plasticity hypothesis

The pathogenesis of depression is significantly influenced by neuroplasticity and remodeling failure (Wang et al., 2021a). The neurotrophic family includes brain-derived neurotrophic factor (BDNF), which regulates neuronal plasticity (Castrén and Monteggia, 2021). On the one hand, it might have an impact on the development of synaptic structures, such as axons and dendrites, and their growth and remodeling. On the other hand, it might enhance the long-term synaptic transmission function of the hippocampus through pre- and post-synaptic pathways (Wardle and Poo, 2003). Decreased levels of BDNF have been found in brain samples from depressed patients (Chen et al., 2001; Dwivedi, 2009; Dwivedi et al., 2009; Carlino et al., 2013). In contrast, antidepressant treatment increases the expression of BDNF in the brains of depressed patients (Chen et al., 2001). Therefore, it shows great potential in the treatment of depression as it increases BDNF levels.

### Neuroinflammatory hypothesis

Glial cells and cytokines play a role in the immune response known as neuroinflammation that occurs in the central nervous system (Zhou et al., 2022). Depression is an inflammation-related disease that worsens as inflammation increases and progresses (Yirmiya et al., 2015; Wang et al., 2021b). According to research, aberrant glial cell activation in the brains of depressed patients results in the release of pro-inflammatory cytokines such as interleukin-6 (IL-6), interleukin 1 beta (IL-1β), and tumor necrosis factor-alpha (TNF-α), which can lead to neuroinflammation and neuronal death (Matraszek-Gawron et al., 2019). In addition, glial cells produce nitric oxide synthase (NOS), and cyclooxygenase-2 (COX-2) could also induce neuroinflammation by promoting oxidative stress levels. Neuroinflammation has emerged as a novel target for the treatment of depression (Wang et al., 2021b; Zhou et al., 2022).

### Hypothalamic-pituitary-adrenal (HPA) axis

The hypothalamus, pituitary, and adrenal glands work together as part of the HPA axis to govern the body’s reaction to physiological or psychological stimuli (Herman et al., 2016; Lew et al., 2020). Patients with depression have been discovered to have HPA dysfunction, resulting in high glucocorticoid (GC) levels, which in turn cause neuronal dysfunction and structural changes in the hippocampus (Dean and Keshavan, 2017). Clinical trials have established the antidepressant properties of glucocorticoid receptor (GR) antagonists and the viability of targeting HPA regulation in the treatment of depression (Dean and Keshavan, 2017).

### MNPs have anti-depressant potential

A significant number of MNPs are produced by the complex living environment of marine animals, and a novel entity structure and enormous diversity are provided by the chemical structure with strong biological activity (Zhao et al., 2022a). Some MNPs have participated in antidepressant preclinical or clinical trials and have proven to be great sources for novel and effective antidepressants (Tables 1, 2). The marine environment is a rich source of novel pharmaceuticals, many of the substances found there can regulate brain activity, reduce anxiety, and have potential therapeutic applications for disorders associated with anxiety and depression (Zhao et al., 2022a). At present, with the interaction between academia and the pharmaceutical sector, a large number of MNPs have been discovered and tested using current analytical methods.

**TABLE 1:** Antidepressant Natural Products of Marine animal Origin.

Natural products	Model establishment	Behavioral outcome	Mechanism	Ref.
Tilapia skin peptides	CP	FST, TST, OFT	Decreased Iba-1, TNF-α, and IL-1β levels	Zhao et al. (2022a)
			Increased Keap1, SOD, and GSH-Px levels via the Nrf2/HO-1 pathway	
			Increased Bcl-2, Bax, and caspase-3 levels via the BDNF/TrkB/CREB pathway	
Bryostatin-1	CUMS	Open water swimming	N.A.	Alkon et al. (2017)
EPA and DHA	CUMS	SPT, TST, OFT	Decreased IL-1β, IL-6, TNF-α, and CD11b levels	Peng et al. (2020)
			Increased BDNF, GDNF and NGF levels	
Resolvin D1	myocardial infarction	FST	Anti-inflammation	Gilbert et al. (2014)
Krill oil	CLET	FST	Increased BDNF levels	Wibrand et al. (2013)
5,6-Br-DMT	FST	FST, TST	N.A.	Zhang et al. (2023)
Astaxanthin	LPS	FST, TST	Decreased TNF-α, IL-1β, IL-6, iNOS, nNOS and COX-2 levels via the NF-κB pathway	Jiang et al. (2016), Jiang et al. (2017)

EPA, eicosapentaenoic acid; DHA, docosahexaenoic acid; 5,6-Br-DMT, 5,6-dibromo-N, N-dimethyltryptamine; CP, cyclophosphamide; CUMS, chronic unpredictable mild stress; CLET, conditioned light extinction test; LPS, lipopolysaccharides; FST, forced swim test; TST, tail suspension test; OFT, open field test; N.A., not applicable.

**TABLE 2:** Antidepressant compounds derived from marine plants.

Natural products	Model establishment	Behavioral outcome	Mechanism	Ref.
Spirulina	FST	FST	Increased serum BUN and LDH levels	Kim et al. (2008)
Neoechinulin A	LPS	FST	N.A.	Sasaki-Hamada et al. (2016)
Zeaxanthin	diabetic rats	OFT, FST	Decreased IL-6, IL-1β and TNF-α levels	Peng et al. (2020)
Fucoxanthin	LPS	FST, TST	Decreased TNF-α, IL-1β, IL-6, iNOS, and COX-2 level via the AMPK/NF-κB pathway	Gilbert et al. (2014)
Lutein	corticosterone	TST, OFT	Decreased corticosterone levels	Wibrand et al. (2013)
β-Carotene	FST	FST	Decreased TNF-α and IL-6 levels	Kim et al. (2016)
			Increased DNF and p-ERK levels	
Total Sterols and β-sitosterol	FST and TST	FST, TST	Increased NE, 5-HT, and the metabolite 5-HIAA	Zhao et al. (2016)
Fucoidan	LPS and CRS	FST, TST	Inhibited caspase-1 levels	Li et al. (2020)
			Increased BDNF levels	
Fucosterol	FST and TST	FST, TST	Increased NE and 5-HT levels	Zhen et al. (2015)
Alternanthera philoxeroides	ovariectomized	FST, TST	Inhibited MAO-A and MAO-B with IC50 values of 252.9 and 90.69 μg/mL, respectively	Khamphukdee et al. (2018)
Botryococcus braunii	FST	FST	Increased BDNF, TH, and PC levels	Sasaki et al. (2017)
Padina australis	corticosterone	N.A.	Decreased corticosterone levels	Subermaniam et al. (2020)
Ulva species	FST	FST	N.A.	Violle et al. (2018)
Chlorella vulgaris	patients	N.A.	N.A.	Jiang et al. (2016), Jiang et al. (2017)
				Panahi et al. (2015)

LPS, lipopolysaccharides; CRS, chronic restraint stress; FST, forced swim test; OFT, open field test; TST, tail suspension test; N.A., not applicable.

### Antidepressant natural products of marine animal origin

#### Tilapia skin peptides (TSP)

Tilapia skin peptides (TSP), derived from tilapia (*Oreochromis mossambicus*) scraps (Zhao et al., 2022a), have biological actions that are antioxidant, anti-inflammatory, anti-apoptotic and hypotensive (Ling et al., 2018; Zhao et al., 2022b; Song et al., 2022). Hippocampal neurons in mice that received an intraperitoneal infusion of cyclophosphamide (CP) experienced oxidative stress, neuroinflammation, and neuronal death (Iqubal et al., 2019). TSP [1,000 mg/kg/d, intragastrically (*i.g.*)] could improve CP-induced depressive-like behaviors such as Sucrose Preference Test (SPT), Forced Swim Test (FST), Tail Suspension Test (TST) and Open Field Test (OFT) in mice (Zhao et al., 2022a). Mechanistically, TSP may reduce CP-induced neuroinflammation by decreasing the expression of ionized calcium-binding adaptor molecule 1 (Iba-1), TNF-α, and IL-1β in the hippocampus of mice. TSP also attenuated CP-induced oxidative stress by increasing the Nrf2/HO-1 signaling pathway and increasing the levels of kelch-like ECH-associated protein 1 (Keap1), superoxide dismutase (SOD), glutathione peroxidase (GSH-Px) and malondialdehyde (MDA). In addition, TSP could also reduce neuronal apoptosis by increasing Bcl-2/Bax/Caspase-3 through the BDNF/TrkB/CREB signaling pathway. In conclusion, the antidepressant effect of the TSP may be involved in the regulation of synaptic plasticity and anti-inflammatory activity (Zhao et al., 2022a).

#### Bryostatin-1

Bugula neritina-derived bryostatin-1 can increase the expression of protein kinase C (PKC), induce PKC membrane translocation, and enhance synaptic plasticity (Nelson et al., 2017). PKC activity was reduced in the brains of depressed individuals (Pandey et al., 2021). In the rat model of depression generated by chronic unpredictable mild stress (CUMS), PKC expression was markedly reduced (Han et al., 2015). According to these investigations, PKC levels and the development of depression may be related. Furthermore, PKC has the ability to significantly alter synaptic transmission (Wierda et al., 2007; Shen et al., 2021). Bryostatin-1 [100 nmol/kg, intravenous (*i.v.*)] shortened the immobility time in the FST in rats. This antidepressant effect of Bryostatin-1 is largely abolished by 1-(5-isoquinolinylsulfonyl)-2-methylpiperazine (H-7), a PKC inhibitor, which suggests that Bryostatin-1 may have an antidepressant effect by enhancing synaptic plasticity through activation of PKC action (Alkon et al., 2017).

#### Eicosapentaenoic acid (EPA) and docosahexaenoic acid (DHA)

Eicosapentaenoic acid (EPA) and docosahexaenoic acid (DHA) are mainly derived from the Omega-3 long-chain polyunsaturated fatty acids (n-3 PUFA) of deep-sea fish and shrimp, which play a key role in brain development (Andraka et al., 2020). Continuous oral administration (*p.o.*) of EPA or DHA for 45 days reduced the weight loss and depressive-like behavior caused by CUMS in SPT/OFT/FST. Mechanistically, EPA and DHA effectively reduced CUMS-induced expression of IL-1β, IL-6, TNF-α, microglial marker α M Integrin alpha M (CD11b), and increased expression of astrocyte marker glial fibrillary acidic protein (GFAP) by regulating the NF-κB/p38 signaling pathway (Peng et al., 2020). Additionally, EPA and DHA modulated the BDNF/TrkB signaling pathway to upregulate the production of BDNF, glial cell-derived neurotrophic factor (GDNF), nerve growth factor (NGF), and Bcl-2 and reduce the expression of Bax, reversing the effects of CUMS-induced neurotrophic factor deficiency and apoptosis (Peng et al., 2020). Moreover, EPA and DHA can also reduce the serum total cholesterol (STC) contents of serum total cholesterol and corticosterone (a glucocorticoid), and induce 5-HT and NE deficiency in the hippocampus, suggesting that EPA and DHA exert antidepressant activity by regulating HPA (Peng et al., 2020). Notably, EPA was more effective than DHA in reducing depressive-like behavior, which was also confirmed in clinical studies. Lipopolysaccharide (LPS) activates BV2 microglia, and docosapentaenoic acid (DPA, a PUFA) balances microglial M1 and M2 polarization, inhibiting NF-κB and p38 while activating neuronal BDNF/TrkB-PI3K/AKT pathways to protect neurons from neuroinflammatory damage (Liu et al., 2021). By increasing the expression of DA, decreasing the expression of NE and gamma-aminobutyric acid (GABA), and reducing the turnover rate of 5-HT in the mouse hippocampus, PUFAs may also enhance CUMS-induced depressive-like behaviors in the SPT, OFT, and FST (Yang et al., 2019). More importantly, a previous clinical trial has been carried out as a result of the positive safety and antidepressant characteristics of EPA and DHA (Su et al., 2014). Nobody left the study during the 2 weeks due to adverse events and, as determined by the investigators, the incidence of Interferon-alpha (IFN-α)-induced depression in patients with hepatitis C virus infection was significantly lower in those treated with EPA but not in those treated with DHA (Su et al., 2014). In a population-based study to prevent the risk of *postpartum* depression in Brazilian pregnant women, a daily intake of 1.8 g of PUFAs (1.08 g of EPA and 0.72 g of DHA) for 16 weeks starting at 22–24 weeks of gestation had no significant effect on early depressive symptoms during pregnancy or *postpartum* (Vaz et al., 2017). However, the Edinburgh Postnatal Depression Scale (EPDS) scores of women in the EPA/DHA group with a history of depression showed a greater decrease from the second trimester to the *postpartum* period. Additionally, there were no changes between the EPA/DHA groups and control groups in terms of gestational duration or birth weight (Vaz et al., 2017). According to a recent meta-analysis, both EPA and DHA have antidepressant effects, although EPA’s are more potent (Sublette et al., 2011).

#### Resolvin D1

Resolvin D1, a PUFA metabolite mostly found in deep-sea fish and shrimp, is effective in reducing inflammation by activating Akt and binding to 2 G-protein-coupled receptors (ALX and GPR32) (Serhan and Chiang, 2008; Nelson et al., 2014). Resolvin D1 reduces the depressive-like behavior seen in experimental models of myocardial infarction when administered before ischemia or 5 minutes after reperfusion (Gilbert et al., 2014). In the FST, there was a statistically significant relationship between infarct size, and immobility time (Gilbert et al., 2014). After myocardial infarction, inflammation is indeed well-documented, especially in the first hours of reperfusion (Sharma and Das, 1997; Nah and Rhee, 2009). Therefore, the anti-inflammatory effect may be the reason for its antidepressant-like function.

#### Krill oil

Antarctic krill (*Euphausia superba*), a zooplankton that resembles shrimp and is rich in EPA, DHA, and astaxanthin, is used to produce krill oil (Wibrand et al., 2013). The conditioned Light Extinction Test (CLET) - induced depressive-like behavior in the FST was reduced in rats after 7 weeks of krill oil administration. Additionally, krill oil reduced depressive-like behaviors by modifying the expression levels of synaptic plasticity-related genes in the prefrontal cortex and hippocampus (Wibrand et al., 2013). Moreover, krill oil supplementation in mice ameliorated chronic unpredictable mild stress (CUMS)-induced depressive-like behaviors by prompting the metabolism of glycerophospholipids and sphingolipids through regulation of differentially expressed genes mainly enriched in the membrane structures and neuroactive ligand-receptor interaction pathway (Zhang et al., 2023). Additionally, Krill oil facilitated fear extinction and reduced depressive-like behaviors by increasing hippocampal calcineurin A levels in mice (Alvarez-Ricartes et al., 2018).

#### 5,6-dibromo-N, N-dimethyltryptamine (5,6-Br-DMT)

5,6-dibromo-N, N-dimethyltryptamine (5,6-Br-DMT) was isolated as a pale light yellow crystal. The precise mechanism underlying how 5,6-Br-DMT [20 mg/kg, intraperitoneally, (*i.p.*)] ameliorated depressive-like behaviors in the FST and TST in mice has not been determined. Indole alkaloids related to 5,6-Br-DMT have been found to have a strong affinity for 5-HT_2_ receptors, indicating that their antidepressant effects may be caused by inhibition of 5-HT reuptake (Hu et al., 2002).

#### Astaxanthin

The red carotenoid pigment astaxanthin is abundant in microalgae, salmon, trout, and marine invertebrates (Ávila-Román et al., 2021). It has numerous pharmacological properties, such as anti-inflammatory and antioxidant activities (Wu et al., 2015; Balietti et al., 2016). Trans-astaxanthin (20–80 mg/kg, *p.o.*) for 7 days prevented mice from displaying depressive-like symptoms after being exposed to LPS (Jiang et al., 2016). Neurochemical analysis showed that trans-astaxanthin could also reverse LPS-induced overexpression of IL-1β, IL-6, and TNF-α, and reduce the expression of inducible nitric oxide synthase (iNOS), neuronal nitric oxide synthase (nNOS), and COX-2 by modulating the NF-κB pathway (Jiang et al., 2016). In conclusion, trans-astaxanthin may produce antidepressant effects through its potent anti-inflammatory properties (Jiang et al., 2016). Similarly, administration of astaxanthin (20–80 mg/kg, *i.g.*) to mice improved their depressive-like behavior and reduced immobility time during the FST and TST (Jiang et al., 2017). Pretreatment with para-chlorophenylalanine (PCPA) (a 5-HT synthesis inhibitor) abolished the anti-immobility effect of Astaxanthin in FST and TST, suggesting that the mechanism of the antidepressant-like effects of Astaxanthin may involve the 5-HT system (Jiang et al., 2017). More importantly, a clinical trial investigated the effects of Astaxanthin on 28 adults diagnosed with depression and fatigue. The study also recruited healthy, active, and non-depressed adults. Subjects who received 12 mg of Astaxanthin daily for 8 weeks significantly reduced depression and fatigue, compared to the group who received a matching placebo (Talbott et al., 2019).

### Natural antidepressants derived from marine plants

#### Spirulina

Spirulina is a kind of true filamentous spiral cyanobacteria protoplasm that has the biological activities of enhancing immunity, antioxidation, reducing cholesterol levels, and relieving hyperlipidemia (Kim et al., 2008; Subermaniam et al., 2021b). Hydrolyzed Spirulina by malted barley reduces immobility time on FST in mice and increases serum blood urea nitrogen (BUN) and LDH levels (Kim et al., 2008). Moreover, Spirulina improved adolescent stress-induced anxiety and depressive-like symptoms via oxidative stress and alterations in prefrontal cortex BDNF and 5HT-3 receptors in female rats (Moradi-Kor et al., 2020). The specific mechanism needs to be further explored.

#### Neoechinulin A

*Aspergillus amstelodami* yielded Neoechinulin A, an isoprenyl indole alkaloid with antioxidant, anti-tumor, and anti-apoptotic properties from Aspergillus fumigatus MR2012 from the Red Sea. Neoechinulin A [300 ng/kg, Intracerebroventricularly, (*i.c.v.*)] significantly ameliorated memory decline caused by LPS and restored immobility time in the FST in mice. This effect may be due to modulation of the 5-HT system by direct or indirect action on the 5-HT_1A_ receptor (Sasaki-Hamada et al., 2016).

#### Zeaxanthin

Zeaxanthin, a yellow-orange xanthophyll, has been extracted from the *cyanobacteria Synechocystis sp*. and *Microcystis aeruginosa and* the microalgae *Nannochloropsis oculate* (Lee et al., 2006; Wojtasiewicz and Stoń-Egiert, 2016). Daily oral zeaxanthin administration from weeks 6–19 could reduce depressive-like behaviors in the OFT and FST of diabetic rats. Zeaxanthin administration could also reduce IL-6, IL-1, and TNF-α overproduction, indicating that it has anti-inflammatory characteristics that help minimize depressive-like behaviors in diabetic rats (Zhou et al., 2018a).

#### Fucoxanthin

Fucoxanthin, a natural carotenoid, is abundant in edible brown seaweed and has been shown to have excellent antioxidant, anti-inflammatory, and anti-diabetic effects (Méresse et al., 2020; Bustamam et al., 2021). In the FST and TST of mice, fucoxanthin (200 mg/kg, *i.g.*) significantly reversed LPS-induced depressive-like behaviors (Jiang et al., 2019). Biochemical analysis showed that Fucoxanthin could inhibit LPS-induced overexpression of IL-1β, IL-6, TNF-α, iNOS, and COX-2 in the hippocampus, frontal cortex, and hypothalamus by regulating the AMPK-NF-κB signaling pathway (Jiang et al., 2019).

#### Lutein

Lutein, orange-yellow, is mainly found in microalgae and *Chlorella vulgaris* (Jalali Jivan and Abbasi, 2019; Wang et al., 2020), and has neuroprotective effects (Stringham et al., 2019). In the TST, OFT, and Splash test (ST), lutein (10 mg/kg, *p.o.*) administered once daily for 7 or 21 days significantly reversed corticosterone-induced depressive-like behaviors. This suggests that Lutein may regulate the HPA to exert neuroprotective effects by reducing the level of glucocorticoids (Zeni et al., 2019).

#### β-Carotene

β-Carotene has been extracted mainly from the microalga *Dunaliella salina* (Han et al., 2019). β-Carotene has also been shown to be a potent inhibitor of oxidative stress and inflammation (Zhou et al., 2018b). Oral administration of β-carotene once daily for 28 days significantly reduced immobility time during the FST in mice (Kim et al., 2016). When compared to the control group, β-Carotene significantly reduced the levels of TNF-α and IL-6, and increased the levels of BDNF and pERK (Kim et al., 2016).

#### Total sterols and β-sitosterol

Total Sterols and β-sitosterol have been extracted from *Sargassum horneri*, a brown seaweed found in the Northwestern Pacific Ocean and adjacent seas of Korea, Japan, and China (Zhao et al., 2016). Total steroids and β-sitosterol have been used to treat scrofula, gall, goiter, and edema (Shao et al., 2014; Shao et al., 2015). In both the FST and TST, mice who received total sterols (100–200 mg/kg, *p.o.*) and β-sitosterol (10–30 mg/kg, *i.p.*) had significantly shorter immobility times. Additionally, NE, 5-HT, and the metabolite of 5-Hydroxyindoleacetic acid (5-HIAA) were all considerably elevated by total sterols and β-sitosterol in the mouse brain, suggesting that these neurotransmitters may be involved in mediating the antidepressant-like function (Zhao et al., 2016).

#### Fucoidan

Fucoidan is a bioactive sulfated polysaccharide abundant in brown seaweed with anti-inflammatory activity (Li et al., 2017). Fucoidan (50–100 mg/kg, *p.o.*) significantly attenuated LPS and chronic restraint stress (CRS) induced depressive-like behaviors in the TST and FST in mice (Li et al., 2020). Fucoidan also reduced the downregulation of BDNF-dependent synaptic plasticity in the mouse hippocampus and decreased caspase-1-mediated inflammation (Li et al., 2020). Furthermore, blocking BDNF abolished the antidepressant-like effects of fucoidan in mice, indicating that fucoidan ameliorates depression by inhibiting inflammation and modulating synaptic plasticity (Li et al., 2020).

#### Fucosterol

Fucosterol is a bioactive compound belonging to the sterol group that can be isolated from algae, seaweed and diatoms (Meinita et al., 2021). Fucosterol exhibits various biological activities including anticancer, anti-inflammatory, anti-neurological, and antioxidant characteristics (Lee et al., 2003; Jung et al., 2013; Gan et al., 2019). Fucosterol (10–40 mg/kg, *i.p.*) significantly shortened the immobility time in the FST and TST of mice. The expression of NE and 5-HT was strongly upregulated by fucosterol in the mouse brain, suggesting that fucosterol may act via these neurotransmitters (Zhen et al., 2015).

#### Alternanthera philoxeroides

*Alternanthera philoxeroides* is a true puree of filamentous, spiral-shaped, blue-green freshwater microalgae (Kim et al., 2008). The crude ethanolic extract of *A. philoxeroides* (250–500 mg/kg, *p.o.* once daily for 8 weeks) significantly ameliorated antidepressant-like behaviors in the FST and TST of ovariectomized mice (Khamphukdee et al., 2018). Additionally, it was discovered that the crude extract controlled the levels of BDNF in the frontal cortex and hippocampus. In addition, the crude ethanol extract of *A. philoxeroides* was found to inhibit both MAO-A and MAO-B with IC_50_ values of 252.9 and 90.69 μg/mL, respectively. These findings suggest that the antidepressant effect of the *A. philoxeroides* extract may be involved in regulating synaptic plasticity and inhibiting MAO activity (Khamphukdee et al., 2018).

#### Botryococcus braunii

*Botryococcus braunii* is a pyramid-shaped green colonial microalga that contains triterpenes (Cheng et al., 2019). Daily administration of *B. braunii* ethanol extract (100 mg/kg, for 14 days*, p.o.*) ameliorated depressive-like behaviors with decreased immobility in the FST (Sasaki et al., 2017). The administration of *B. braunii* ethanol extract induced upregulation of gene expression associated with energy metabolism (polyribonucleotide nucleotidyltransferase 1/PNPT1), dopamine production (arginine/serine-rich coiled-coil 1/SRC1), and neurogenesis (short stature homeobox 2/SHOX2, paired-like homeodomain transcription factor 2/PITX2, teashirt zinc finger family member 1/TSHZ1, LIM homeobox 9/LHX9). In addition, the expression of BDNF, tyrosine 3-monooxygenase (TH), and pyruvate carboxylase (PC) was also upregulated (Sasaki et al., 2017). The antidepressant effect of *B. braunii* in animal models of depression is mediated by enhancing energy promotion, neurogenesis, and dopamine synthesis in the brain.

#### Padina australis

*Padina australis* is a species of brown macroalgae belonging to the class Phaeophyceae (Subermaniam et al., 2021a). *P. australis* has been reported to possess numerous biological activities including antioxidant, anti-neuroinflammatory, and anti-acetylcholinesterase properties (Gany et al., 2014). pretreatment with *P. australis* (0.25 mg/mL) attenuated high-dose corticosterone-mediated oxidative damage in a PC12 cell model mimicking depression (Subermaniam et al., 2020). *P. australis* reversed the effects of corticosterone, which decreased cell viability, glutathione levels, aconitase activity, and mitochondrial membrane potential while increasing the release of lactate dehydrogenase. This finding indicates that *P. australis* could be developed as a mitochondria-targeted antioxidant to mitigate antidepressant-like effects (Subermaniam et al., 2020).

#### Ulva species

*Ulva species* are green macroalgae found in marine, fresh, and brackish waters. *U. species* are widely distributed throughout the world with 18 species identified in Japan (Shimada et al., 2008). Acute and subchronic oral toxicity studies showed that 10–40 mg/kg body weight/day of hydrophilic extract of *U. species* for 14 days significantly reduced the immobility time in the FST in rats (Violle et al., 2018). *U. species* have the potential to be a useful supplement or replacement for currently prescribed antidepressants. Further studies are necessary to confirm the mechanism of action of MSP and its modulation of brain function (Violle et al., 2018).

#### Chlorella vulgaris

*Chlorella vulgaris* is a unicellular green microalgae with many pharmacological properties that include antioxidant, anti-inflammatory, antihypertensive, detoxifying, and anti-atherosclerotic effects (Panahi et al., 2012a; Panahi et al., 2012b). A clinical trial investigated the effects of *C. vulgaris Beijerinck* on 92 patients with major depression. 42 patients were assigned to adjuvant therapy with *C. vulgaris*, while 50 patients received standard antidepressant therapy. Participants in the *C. vulgaris* intervention group received six 300 mg tablets per day for 6 weeks, and the intervention group showed improvements in somatic and cognitive symptoms of depression and anxiety (Panahi et al., 2015).

#### Current regulatory situation and commercialization of MNPs

MNPs are the source of modern marine pharmaceuticals. The study of MNPs, which originated in the 1930 s, can be regarded as the starting point of modern marine drug research. So far, about 33,200 new MNPs have been reported. Based on these new MNPs, the FDA has approved eight marine drugs, i.e., Cefalotin, Alexan, Zikonotide, Omega-3 fatty acid ethyl ester, Ericline mesylate, Brentuximab vedotin, and Trabectedin. Research on MNPs in China began in the 1970 s. In China, the first Marine Pharmaceutical Symposium was held in 1979. In 1982, the journal “Chinese Marine Drugs” was founded. In 1985, the first marine polysaccharide new drug, alginate diester sodium (for cardiovascular disease), was successfully developed and approved for marketing in China in 1990. In view of the unique structure and significant activity of MNPs, the Ministry of Science and Technology launched the Marine “863” Science and Technology Project (“863” Marine Biotechnology Research Program) in 1996. The National Natural Science Foundation of China also separated marine drugs from medicinal chemistry and funded them separately in 2008. These initiatives have greatly promoted the development of marine natural products in China and trained a group of excellent marine drug researchers. So far, about 6,700 new MNPs have been found in China, accounting for approximately 20% of the world’s new MNPs.

## Discussion

With the continuous development of modern society, the incidence of depression is increasing, but the existing antidepressant drugs are not effective enough to meet the clinical needs. Therefore, the need for novel, effective antidepressant treatments is critical. In total, 95% of biodiversity and 71% of the Earth’s surface are in the oceans (Haefner, 2003; Sagar et al., 2010). The physical and chemical conditions of the ocean provide marine organisms with unique active compounds that offer new possibilities for the development of new drugs. The data presented in this review shows the great value of MNPs and their derivatives in the prevention and treatment of depression, demonstrating the potential of MNPs as a promising source of antidepressant drugs. Through a variety of processes, such as the modulation of neurotransmitter systems, synaptic plasticity, anti-inflammatory qualities, and the modulation of HPA function, these MNPs exhibit antidepressant properties. However, most of the current efficacy of MNPs and derivatives in the treatment of depression is based on data from *in vitro* and *in vivo* studies, and a large number of clinical studies are still needed to prove their safety and efficacy, which will help to develop promising new medicines. With the in-depth exploration of marine organisms by mankind, an increasing number of new compounds will be continuously extracted and isolated from marine organisms, which will bring new impetus to the treatment of depression, a disease that plagues the world.
